# Case of Nephritic Colic, with Remarks

**Published:** 1855-09

**Authors:** Theo. P. Mayo

**Affiliations:** Demonstrator of Anatomy in the Medical College of Virginia


*Case of Nephritic Colic, with Remarks.* By THEO. P. MAYO, M. D., Demonstrator of Anatomy in the Medical College of Virginia.

Reports of marvellous cases are always interesting, and sometimes elicit discussions and inquiry which result in great benefit to medical science. They ought, however, to be taken with many grains of allowance, except where they emanate from undoubted authority.

We sometimes read and hear of remarkable instances of gun-shot wounds—such as a ball entering on one side of the body, passing entirely around and making its exit at the point of entrance—of patients relieved in divers ways of a large quantity of brains, and recovering with the functions of innervation unimpaired. We have very lately read of consumptives with scarcely any lungs, being swabbed out and perfectly cured; in fact we might be almost tempted to believe that by a judicious combination of sponge and nitrate of silver, syringes and iodine, these organs could be manufactured to order. I myself have seen a case which was under the charge of a practitioner in this city, where about one-half of the parietal bone of one side was knocked off—the brain suppurated considerably, and yet the patient recovered.

The case below might be more properly enumerated under the head of what our French friends would designate as *outre*, and is reported more for the purpose of eliciting from some of our older and more learned brethren their opinion as to its possibility.

Roy, a negro man, æt. 24, entered the Infirmary of the Medical College of Virginia on the 15th of June, 1854. He had contracted gonorrhœa, or, as he termed it, "runnin reins," some eighteen months before, and had as a concomitant to the disease, swelled testicle.

When he entered the wards of the hospital there was a slight gleety discharge from the urethra, and the testicle, which had previously been enlarged, was very much atrophied, being about the size of a filbert; the scrotum presenting the appearance and feel of a serous cyst. This shrunken condition of the testis was evidently distressing Roy very much, and his anxiety for relief was strongly manifested.

After some days he was either really or apparently attacked with severe pain in the right kidney, and along the course of the ureter on that side. In conjunction with these symptoms he had fever and headache.

Fifteen hours after the commencement of this attack, while attempting to urinate, he passed a round body about the size of a buck-shot, which upon examination was found to be *iron.* He stated that he heard it strike against the vessel when he was voiding his water.

About three weeks afterwards, having had no return of colicky symptoms and being entirely relieved of the gleet, he was discharged as well.

I lost sight of him for about a fortnight, when he called and brought me two other bodies, which, according to his statement, he had passed from the urethra; the voiding of which was preceded by symptoms precisely similar to those in the former case. These two specimens were undoubtedly ordinary *leaden shot.*

Just so soon as I found out the nature of these bodies, I stripped him and made a thorough examination, with a view to ascertain whether he had ever been the recipient of a gun-shot wound in the lumbar region, but could find no evidence of that, either there or upon any other portion of his body. The thought then struck me that perhaps the first body might have been the extremity of a bougie or Lallemande's port-caustique, or some other thing of the kind, but upon investigation learned that no instrument of any kind had ever been introduced into his urethra. I inquired but could not ascertain whether he had taken internally any preparation of lead or iron.

My own opinion is that it is one of those cases similar to those deceptive ones we so often meet with in hysterical females. That males are sometimes affected with the disease termed hysteria, is I believe, an universally conceded point.

All of us have heard of, and many seen, cases where some women, with a predisposition to hysteria, insert cinders and other foreign substances into the vagina; whilst others are affected with symptoms of catalepsy, and others again, with those of peritonitis, &c., &c., in fact, so varied and diversified are the cases of hysteria, that the disease is termed Protean.

This boy was a great buck among the dark damsels, and so far as I

can learn had some serious notions of matrimony; and the fact of his testicles "drying up," as he expressed it, might have had such an effect upon his nervous system, as to produce these hysterical symptoms. Why might not he, laboring under that disease (hysteria) have *apparently* a fit of gravel, as well as women in the same supposed condition, be affected with counterfeit symptoms of catalepsy, peritonitis, and a host of other ills?

But my object is one more particularly of inquiry concerning the two following points:

1st. Whether such bodies have ever been passed from the bladder except as nuclei of calculi? for these were entirely devoid of any incrustation.

2nd. Whether the administration of the metals or their salts, and particularly *iron and lead*, either by the stomach, or as injections by the urethra, has ever been known to result in the formation of deposits of the pure metal in the bladder?

For an answer to my first inquiry I must call on Dr. Gross, who has lately published the most extensive and accurate work on diseases of the urinary organs ever written; and upon all the other brethren of the profession who have paid any attention to, or had any experience in, those affections.

To the second query, I would solicit a response from the physiologists and chemists, who may possibly be able to construct a theory which will satisfy our minds with regard to this curious matter.—*Virginia Medical and Surgical Journal.*

